# Supercritical Carbon Dioxide Extraction of Coumarins from the Aerial Parts of *Pterocaulon polystachyum*

**DOI:** 10.3390/molecules29122741

**Published:** 2024-06-08

**Authors:** Júlia M. Scopel, Bruna Medeiros-Neves, Helder Ferreira Teixeira, Nathalya T. Brazil, Sérgio A. L. Bordignon, Fernando Mendonça Diz, Fernanda Bueno Morrone, Rafael N. Almeida, Eduardo Cassel, Gilsane L. von Poser, Rubem M. F. Vargas

**Affiliations:** 1 Unit Operations Laboratory (LOPE), School of Technology, Pontifical Catholic University of Rio Grande do Sul, Av Ipiranga 6681, Building 30, Block F, Room 208, Porto Alegre 90619-900, RS, Brazil; juliamscopel@gmail.com (J.M.S.); rnolibos@gmail.com (R.N.A.); cassel@pucrs.br (E.C.); 2 Programa de Pós-Graduação em Ciências Farmacêuticas, Universidade Federal do Rio Grande do Sul, Porto Alegre 90010-150, RS, Brazil; bruna.medeiros@ufrgs.br (B.M.-N.); helder.teixeira@ufrgs.br (H.F.T.); nathalya.brazil@ufrgs.br (N.T.B.); bordignon@ibest.com.br (S.A.L.B.); gilsane.von@ufrgs.br (G.L.v.P.); 3 Programa de Pós-Graduação em Medicina e Ciências da Saúde, Pontifícia Universidade Católica do Rio Grande do Sul, Porto Alegre 90619-900, RS, Brazil; fernandomendoncadiz@gmail.com (F.M.D.); fernanda.morrone@pucrs.br (F.B.M.)

**Keywords:** *Pterocaulon polystachyum*, supercritical fluid extraction, coumarin, mathematical modeling, cell viability assay

## Abstract

*Pterocaulon polystachyum* is a species of pharmacological interest for providing volatile and non-volatile extracts with antifungal and amebicidal properties. The biological activities of non-volatile extracts may be related to the presence of coumarins, a promising group of secondary metabolites. In the present study, leaves and inflorescences previously used for the extraction of essential oils instead of being disposed of were subjected to extraction with supercritical CO_2_ after pretreatment with microwaves. An experimental design was followed to seek the best extraction condition with the objective function being the maximum total extract. Pressure and temperature were statistically significant factors, and the optimal extraction condition was 240 bar, 60 °C, and pretreatment at 30 °C. The applied mathematical models showed good adherence to the experimental data. The extracts obtained by supercritical CO_2_ were analyzed and the presence of coumarins was confirmed. The extract investigated for cytotoxicity against bladder tumor cells (T24) exhibited significant reduction in cell viability at concentrations between 6 and 12 μg/mL. The introduction of green technology, supercritical extraction, in the exploration of *P. polystachyum* as a source of coumarins represents a paradigm shift with regard to previous studies carried out with this species, which used organic solvents. Furthermore, the concept of circular bioeconomy was applied, i.e., the raw material used was the residue of a steam-distillation process. Therefore, the approach used here is in line with the sustainable exploitation of native plants to obtain extracts rich in coumarins with cytotoxic potential against cancer cells.

## 1. Introduction

*Pterocaulon* is a genus consisting of 18 species, some of them used in popular medicine, mainly as infusions and decoctions, for treating skin, liver and respiratory diseases. Coumarins are considered the main active substances responsible for purported benefits in the treatment of certain ailments [1,2].

Previous studies conducted with *Pterocaulon polystachyum* DC showed that this species is rich in coumarin [1,2,3,4] and also produces essential oils. The essential oil obtained by hydrodistillation presents activity against *Acanthamoeba polyphaga*. Furthermore, regarding amebicidal activity, the hexane extract of this plant demonstrates activity against *A. castellanii* [1].

The plant has also been investigated for cytotoxic activity. The petroleum ether extract of *P. polystachyum* tested against human promonocytic U-937 cells reduced proliferation and induced differentiation of proliferation.

The current market for drugs is geared toward obtaining end products through organic synthesis involving solvents that are generally harmful to health and undesirable for the environment. In order to circumvent this issue, green techniques such as supercritical fluid extraction (SFE) began to be widely investigated [5,6,7]. Such techniques have been successfully employed to obtain essential oils [8], flavonoids [9], coumarins [10], and other natural compounds [11,12].

Supercritical extraction is dependent on operating conditions, that is, variables such as pressure, temperature, solvent flow, granulometry of the solid material, and presence of co-solvent, impact the yield and selectivity of the extraction [13]. In this sense, experimental designs that help in understanding and optimizing the response of a system or process have often been used for supercritical extraction [14]. The dependence of the response on factors such as temperature and pressure [15], co-solvent flow [16,17], extraction time [18], and the effects of raw material pretreatment [19] has been investigated. The response variable, depending on the purpose of the investigation, is the process yield [20] or the selectivity of a target component [21].

In addition to identifying suitable conditions for a process, the mathematical modeling of extraction processes is an important procedure used to improve knowledge of the behavior of a process in order to better predict the performance of extraction units in functional dimensions and different operating conditions [22,23]. Different factors have been taken into account while developing mathematical models to depict supercritical extraction. Many processes that employ this technique are based on a bed consisting of the raw material through which the supercritical fluid passes. Therefore, models based on the mass balance in the phases present in the extraction beds have been proposed in the literature [24,25,26] and widely used in the simulation of extractive processes [27,28,29].

Plants of the genus *Pterocaulon* (Asteraceae) have been studied by our research group, and the presence of coumarins in the *P. balansae* and *P. lorentzii* SFE extracts was evinced [30,31].

Considering that both essential oil and coumarins exhibit relevant activities, and following current trends in developing technologies intended to maximize the use of natural resources and reduce waste, the aim of this study was to take full advantage of the aerial parts of *P. polystachyum*, previously subjected to extraction of essential oil by hydrodistillation.

The present study was designed to maximize the total extract yield of non-volatile compounds from the aerial parts of *P. polystachyum* by investigating the influence of extractive process variables such as pretreatment by microwaves and pressure and temperature of the supercritical fluid. Mathematical modeling was carried out to generate information that could assist in the design of supercritical coumarin extraction units. Finally, the extracts obtained were evaluated for the presence of coumarins and cytotoxic activity against bladder tumor cells (T24).

## 2. Results

### 2.1. Extraction and Response Surface Method

The results of the experiments are showed as Supplementary Material and the response surface were generated by Minitab^®^ 19.1. The symbols PE, TE, and TP stand for extraction pressure, extraction temperature, and microwave pretreatment temperature, respectively. The response variable was the total extract yield.

Initially, a quadratic model was generated, consisting of three linear, three quadratic and three interaction components of pressure, temperature, and pretreatment temperature. This model presented an R^2^ of 88.13%, indicating that it is a good representation of the obtained experimental data. The adjusted R^2^ value was 66.75%, and the factor considered significant (*p* < 0.05) was the linear component of the extraction pressure. On the other hand, the model itself was not considered significant at a significance level of 95% and was not able to predict the response outside the considered region. For these reasons, we chose to use the stepwise regression method to improve the quality of the model. This method consists in adding or removing terms iteratively, aiming to discard terms that are not statistically significant and whose presence may contribute to an increase in error and to impair the model predictive capacity [32,33,34].

The statistical data (ANOVA) exhibited in Table 1 indicate that the stepwise regression model is statistically significant (*p* = 0.006) and inform that the components PE and TE present values of *p* < 0.05. The factors that most contribute to the model are the extraction pressure and temperature variables, responsible for more than 50% of the response. The values for the parameters R^2^ and R^2^adj were 85.08 and 73.89.

According to Myers et al. [35] a high value of R^2^ does not necessarily mean that the regression model is good, since the addition of variables will always increase the value of R^2^, even if the variable is not significant. The same does not occur with the adjusted R^2^ value, which decreases as non-significant terms are added to the model [36,37]. For the stepwise regression model, the adjusted R^2^ was 73.89%, which means that there is a better dependency between the dependent variable (extract yield) and the chosen independent terms (PE, TE, and TP) when compared to the conventional quadratic model. The S-value is another indication of how well the model fits the response obtained experimentally and is measured in the units of the response variable. The smaller it is, the better the response description is. The value of S for the stepwise model was 1.2748, while for the quadratic model it was 1.4384, which corroborates the better quality of the presented stepwise adjustment model. The stepwise regression model generated in non-coded units is expressed by equation 1 for the overall extract yield (%):

(1) $$R\left(\%\right)=43.9-0.0388\times PE-1.927\times TE+0.1027\times TP+0.01465\times {\mathrm{TE}}^{2}\;+0.00290\times PE\times TE-0.000554\times PE\times TP$$

The surfaces generated from the design of experiments and the model generated by regression are presented below. The graphs were created using Statistica 10.0 software and the values of the variables that are not on the graph were maintained at the value corresponding to the central point (PE = 200 bar; TE = 50 °C; TP = 90 °C).

**Figure 1:**
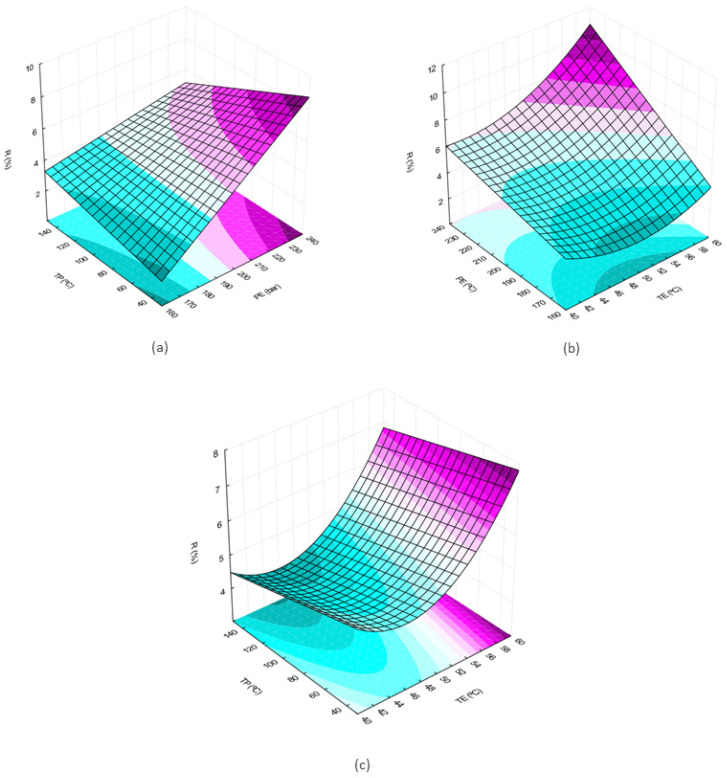
Response surfaces for effects of two independent variables on the global yield of the extract obtained by supercritical extraction: (**a**) extraction pressure (P) and pretreatment temperature (TP); (**b**) extraction temperature (TE) and extraction pressure (P); (**c**) pretreatment temperature (TP) and extraction temperature (TE).

From the observation of the surfaces, it is possible to perceive an increase in the extract yield with the increase in the pressure and temperature variables (Figure 1a–c). In Figure 1a, where the temperature is fixed, it is observed that at the maximum pretreatment temperature, the pressure influence is smaller when compared to the region where the pretreatment temperature is minimum comparing the slopes in the two surface edges. When the extraction pressure is kept constant (Figure 1c) the slope of the response surface is observed in the direction of increasing yield as TP decreases.

The optimal conditions were obtained for the three variables considered in the experimental design using the Minitab^®^ optimization tool. The values obtained were 240 bar and 60 °C for the extraction conditions and 30 °C for the optimal pretreatment condition, data that are consistent with what was observed on the response surfaces presented in Figure 1, as already discussed. The average value of the yield between three experiments can then be compared to the value predicted by the polynomial obtained by regression, as in Equation (1). The average experimental yield was 10.46% and the standard deviation was 2.05%, which corresponds to approximately 0.5 g of extract. The yield calculated by the regression model was 12.56%, a value that is located within the region comprising the experimental deviations.

### 2.2. Mathematical Modeling

**Figure 2:**
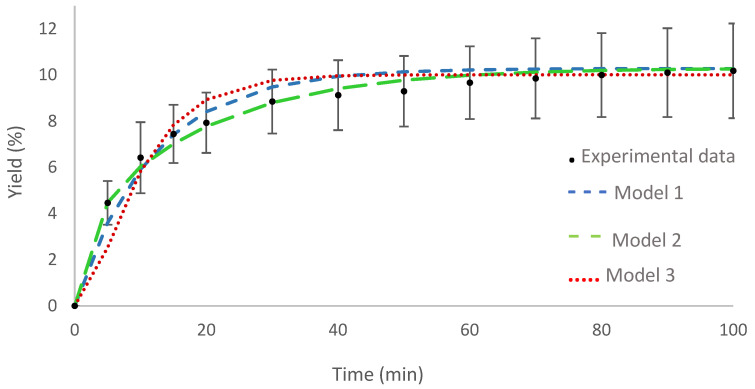
Curve of the optimal condition for supercritical fluid extraction: experimental data and adjustment of mathematical models.

The curve for the optimal condition is shown in Figure 2 together with the adjustment of the proposed mathematical models. The three models showed satisfactory adherence to the experimental data, model 2 being the one that best represents the experimental data, given the high R^2^ value.

Table 2 presents the values for the parameters provided by the mathematical models for the optimal condition of supercritical fluid extraction. The values of the parameters for the diffusion coefficient obtained for Model 1 are similar in magnitude to those reported in the literature for supercritical fluid extraction [38], and also the values found for the surface mass transfer coefficient showed the same order of magnitude found by Almeida et al. [39].

The internal mass transfer coefficient, in Model 2, resulted in an order of magnitude of 10^−8^ m/s. This same order was found by other authors investigating different plant species [40].

The parameter values of Model 3 are relatively high when compared to the values obtained by Almeida et al. [39]. However, Falcão et al. [38] obtained higher values for k_p_, with an order of magnitude of 10^−2^, being closer to that obtained in this work. The relatively high order of magnitude of parameters (10^−3^) obtained for Model 3 may be associated with the high amount of extract and the speed with which it is extracted from the plant matrix. A study by Silva et al. [41] found an order of magnitude of 10^−3^ for the partition coefficient in the same way as in this article.

### 2.3. Analysis of the Extracts Obtained in the Experimental Design

**Figure 3:**
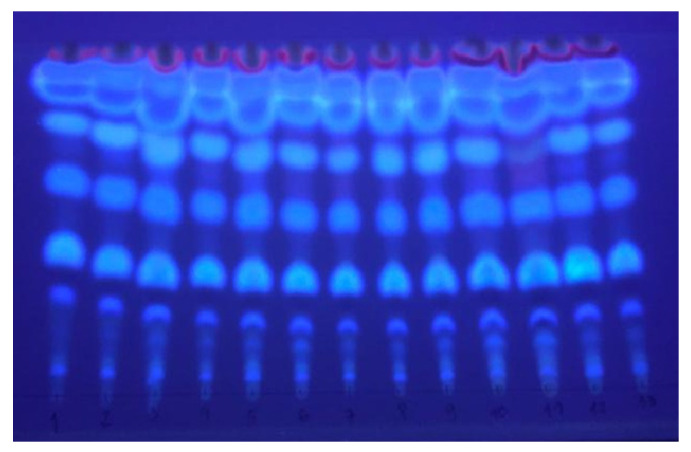
TLC of the 13 different extracts under UV light (365 nm).

The samples obtained in the experimental design were analyzed by thin-layer chromatography (TLC). The chromatogram of the SFE extracts obtained from plant material previously subjected to steam distillation and subsequently exposed to microwaves revealed the presence of several spots with intense blue fluorescence at 365 nm, characteristic of coumarins (Figure 3). *Pterocaulon polystachyum* is a species known for providing extracts rich in coumarins. At least two dozen different coumarins have already been reported for this plant [1].

Due to the similarity presented between the extracts obtained by different experimental design conditions, a single sample was randomly chosen and qualitatively analyzed by ultrafast liquid chromatography (UFLC). The sample analyzed was that obtained at 160 bar, 50 °C, and microwave pretreatment at 150 °C (sample C). Another sample obtained with the same supercritical fluid extraction condition but without microwave exposure and without previous extraction of essential oil (sample A) and another without microwave treatment (sample B) were also analyzed by UFLC to verify the possible influence of pretreatments on the composition of the extract.

Supercritical fluid extraction is an alternative for extraction of thermolabile compounds [42,43]. In this sense, techniques that promote the heating of the vegetal matrix can contribute to the alteration of the chemical composition of the extracts, as is the case with steam distillation and microwaves. For this reason, samples of extracts using the same supercritical fluid extraction condition but which did not pass through the mentioned steps were also analyzed by UFLC.

**Figure 4:**
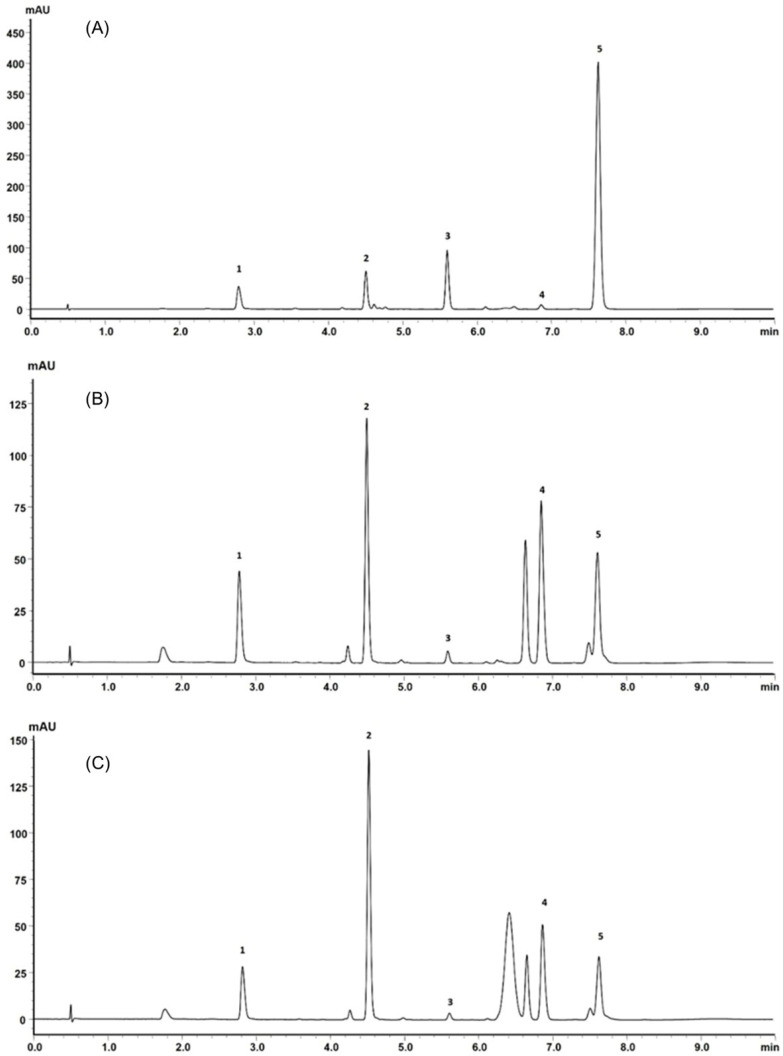
(**A**) UFLC chromatogram profile of the extract obtained from plant material subjected only to SFE, (**B**) SFE extract obtained from plant material previously subjected to steam distillation and (**C**) SFE extract obtained from plant material previously subjected to steam distillation and subsequently exposed to microwaves.

Retention times and UV absorption spectra were compared with those obtained in previous analyzes of other extracts of species of the genus *Pterocaulon* under the same conditions. Except for the compound at the retention time of 6.4 min, in sample C, the chromatograms, from a qualitative point of view, are very alike. In Figure 4, the three samples (A, B, and C) showed peaks with a retention time (Tr) of approximately 2.9 (peak 1) and 4.5 min (peak 2), attributed to 7-(2,3-dihydroxy-3-methylbutyloxy)-6-methoxycoumarin (obtusinin) and 5-methoxy-6,7-methylenedioxy-coumarin, respectively. Comparing these chromatograms with those obtained by Barata-Vallejo [44], it is possible to infer that the peak with Tr of 5.6 min (peak 3) is related to the presence of 7-(2,3-epoxy-3-methylbutyloxy)-6-methoxycoumarin.

**Figure 5:**
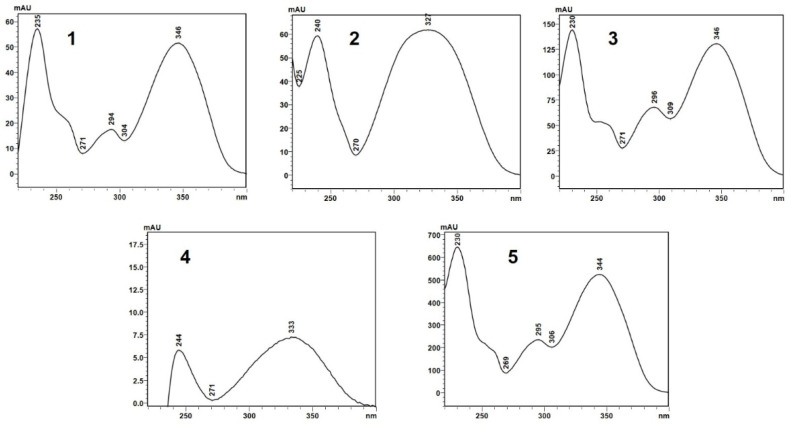
UV absorption spectra (327 nm) of the compounds indicated by peaks 1–5 in the chromatograms shown in Figure 4A–C.

**Figure 6:**
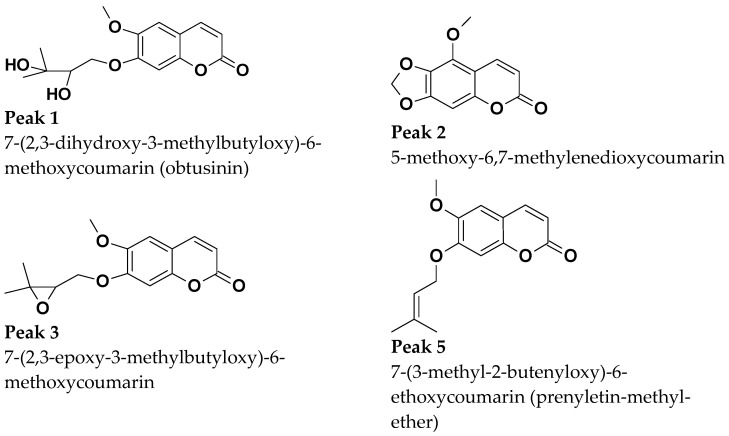
Coumarins characterized shown in Figure 4.

The compound indicated by peak 4, present in a small amount in the supercritical extract of the plant material without prior treatment, could not be characterized. The UV profile is somewhat similar to that of 5-methoxy-6,7-methylenedioxy-coumarin (Figure 5). However, it was impossible to infer its possible structure. Also, based on Barata-Vallejo’s [44] chromatogram, is possible to suggest the presence of 7-(3-methyl-2-butenyloxy)-6-methoxy-coumarin in the three extracts (Rt 7.6 min) (peak 5), since this coumarin was found in the dichloromethane extract of the plant, being one of the major peaks in the chromatogram. To confirm the identity of this compound, the extract obtained from the plant without pretreatment was submitted to column chromatography. The isolated compound was subjected to spectroscopic analysis, which allowed confirmation of the structure of 7-(3-methyl-2-butenyloxy)-6-methoxy-coumarin. This compound, trivially named prenyletin-methyl-ether, has previously been isolated from this plant [1]. The structures of the coumarins are exhibited in Figure 6.

Although the analysis was qualitative, it is possible to verify in the chromatogram that when the plant material was pretreated, there was slightly better selectivity for 5-methoxy-6-7-methylenedioxycoumarin (peak 2), a compound that has already demonstrated activity against glioma cells [45]. In the extract obtained from the plant without pretreatment, the main product is prenyletin-methyl-ether (peak 5), with 5-methoxy- 6-7-methylenedioxycoumarin present in smaller quantities. Therefore, if 5-methoxy-6-7-methylenedioxycoumarin is the target compound, pretreatment methods are indicated, since in addition to it being possible to obtain essential oils, the compound appears in greater quantity.

### 2.4. Cell Viability Analysis (MTT)

**Figure 7:**
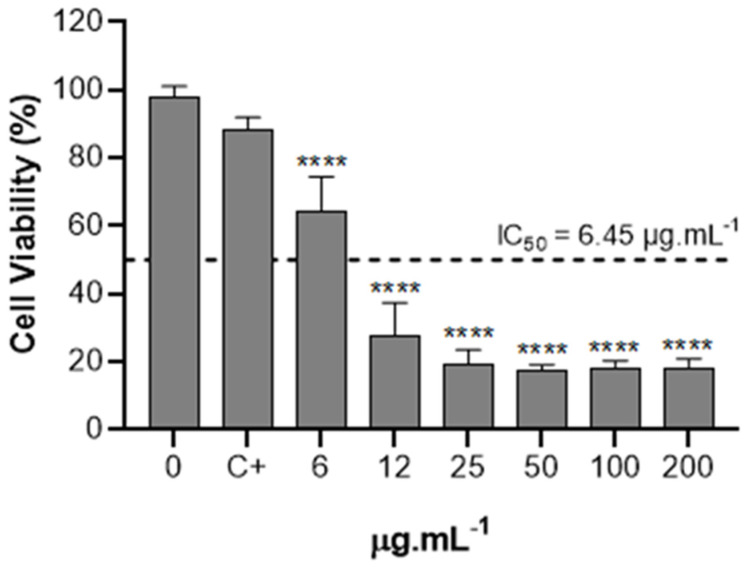
Cytotoxic effect of extract obtained by supercritical CO_2_ on the viability of T24 cell line. At 80-90% of confluence, T24 cells were treated with 6, 12, 25, 50, 100, and 200 µg.mL^−1^ of extract obtained by supercritical CO_2_ for 24 h. The data were analyzed for statistical significance using one-way ANOVA, followed by Tukey’s post hoc test. C+ is the control with the vehicle (ethanol) in which the extract was solubilized. **** *p* < 0.0001.

The effect of the extract obtained through supercritical CO_2_ on the cytotoxicity in bladder tumor cells (T24) was assessed. T24 cells were exposed to increasing concentrations from 6 to 200 µg.mL^−1^ for 24 h, and cell viability is illustrated in Figure 7. It was possible to observe a significant reduction in cell viability between concentrations of 6 and 12 μg/mL. No significant differences were observed among the concentrations of 25, 50, 100, and 200 μg/mL for the extract obtained through supercritical fluid. The IC_50_ values of extracts were calculated using linear regression from cell viability data and extract concentrations, both on a base 10 logarithmic scale [45]. The IC_50_ represents the concentration required to reduce cell viability by 50%. For the extract obtained via supercritical fluid, the calculated IC_50_ value was 6.45 μg/mL.

The cytotoxicity of the analyzed extract is likely associated with the presence of 5-methoxy-6,7-methylenedioxycoumarin. Vianna et al. [45] have already reported the cytotoxicity of this compound in two types of glioma tumor cells using the MTT assay. The tumor cells used in the experiments were human (U138-MG) and mouse (C6) cell lines, with calculated IC_50_ values of 34.6 μM and 31.6 μM, respectively, after 48 h. These values translate to 7.62 and 6.95 μg/mL, respectively, which closely align with the results obtained in this study. Notably, it is worth highlighting that Vianna et al. [45] utilized isolated coumarin, whereas in our research, we employed the total extract obtained through supercritical extraction. The cytotoxicity of this coumarin in leukemic cells of the U-937 lineage has also been documented in a study conducted by Riveiro et al. [4]. Studies conducted by Mongelli et al. [46] indicated the ability of extracts obtained from various medicinal plants to interact with cell DNA. This interaction occurs with many chemotherapy drugs, as noted by Palchaudhuri and Hergenrother [47]. One of the plant species explored in their investigation was *P. polystachyum*, a finding that supports our own results.

## 3. Materials and Methods

### 3.1. Plant Material

Aerial parts of *P. polystachyum* were collected in the summer at Nova Santa Rita, Rio Grande do Sul, Brazil (29°54′01.7″ S 51°16′56.1″ W). The species was identified by Dr. Sérgio Augusto de Loreto Bordignon. A voucher specimen was deposited in the Herbarium of the Botanic Department of the Federal University of Rio Grande do Sul (ICN-UFRGS). After collecting the aerial parts, the leaves and inflorescences were separated from the stems and dried at room temperature, protected from light.

### 3.2. Pretreatment

The plant material previously subjected to essential oil extraction by steam distillation was dried, resulting in a moisture content of 3.11%, and ground in a knife mill, giving particles with an average diameter of 0.24 mm, determined by classification on sieves. Subsequently, the material was submitted to the pretreatment process in microwave equipment (Lab-Kits, model WM-MD6M), with 1200 W power and operating frequency of 2450 MHz. The pretreatment temperatures considered in the experimental planning were defined in order to seek a mild treatment (T < 150 °C). The power was defined as 50% of the total power, and the time in which the sample was kept at the temperature chosen for each pretreatment was 1 min.

### 3.3. Extraction with Supercritical Fluid

The extractions were carried out in the pilot unit of supercritical extraction, a description of which is found in Scopel et al. [48]. The solvent flow rate used in the extractions was 1000 g/h and the extraction time was set at 2 h, enough time for the plant to deplete according to previous experiments. At the end of each extraction, the flasks were weighed to obtain global extract yield data (Figure 1). The experiments were carried out in an extractor vessel with an internal volume of 100 mL and the plant mass used was 23 g. The solvent:feed ratio used was 86.9 g:g. The presence of coumarins in the extracts was determined by thin-layer chromatography (TLC).

### 3.4. Experimental Design for SFE: Box-Behnken Technique

The use of experimental design techniques that help to understand and optimize the response of a system or process has often been used for supercritical extraction [49]. In this study, the experiments were planned using the Box–Behnken technique in Minitab^®^ software, where three factors were evaluated: extraction pressure, extraction temperature, and microwave pretreatment temperature.

Before starting the experiment itself, using microwaves as a pretreatment, the plant material resulting from steam distillation (after drying and grinding) was subjected to a sequential extraction with supercritical CO_2_ following a procedure presented by Torres et al. [33], where a scan of pressure conditions (80, 120, 160, 200, 240, 280, and 300 bar) at a constant temperature of 40 °C was carried out in order to identify adequate pressure conditions to obtain extracts containing coumarins. The extracts resulting from this process were analyzed by TLC. In all the fractions obtained at different pressures, the presence of compounds with strong fluorescence characteristic of coumarins was verified. Therefore, the conditions applied were suitable for obtaining these compounds. Once the appropriate parameters had been established, the extractions following the experimental planning indicated in Table 3 (experimental section) were carried out. The temperature range was chosen based on information from previous work with similar plant species [32,33].

Given that total extract mass values had been obtained in the experiments, Minitab^®^ was used as an optimization tool. Subsequently, for the optimal condition found, the extraction curve was constructed. For this purpose, the flasks were weighed at intervals of 5 min in the first 20 min of extraction and then at intervals of 10 min.

### 3.5. Mathematical Modeling

The mathematical modeling of supercritical extraction is a procedure that has been extensively investigated because it is decisive in monitoring and developing units, whether on a laboratory, pilot, or industrial scale. The aim was to evaluate the performance of three mathematical models supported by different hypotheses in the representation of the extraction process.

Model 1 proposed by Crank [50] assumes that diffusion is the phenomenon that governs mass transfer and is described by Fick’s second law in rectangular, unidimensional coordinates and subject to a convective boundary condition [51]. It considers that the solute is uniformly distributed in the solid particles and that the transfer rate to the fluid surrounding the particle is directly proportional to the difference in solute concentration between the surface of the particle and the fluid that flows externally. In this model, the diffusion coefficient of the solute in the solvent, D, and the surface mass transfer coefficient, k_c_, are unknown and will be determined by the parameter adjustment technique. This type of model has been successfully used by some authors to model supercritical extraction [48,48,52].

Model 2 is based on the proposal made by Sovová [25], which considers the solute uniformly distributed in two types of solid particles, intact and broken. Intact particles are associated with structures with difficult access to solute and ground particles with easy access to solute. Based on these hypotheses, Silva et al. [53] considered extraction with negligible external mass transfer resistance and presented mathematical equations for the mass of extract obtained as a function of time for each of the two stages of the extractive process. The authors [53] added an expression for the time associated with exhaustion of the easily accessible solute and rewrote the model with this consideration. The unknown parameters of this model are the mass transfer coefficient in the solid phase, ks, the solute mass fraction in the fluid phase at the saturation condition, Y*, the mass of easily accessible solute, M*, and time to start the extraction of difficult-to-access solute, τ.

Model 3, proposed by Reverchon [24], is based on the differential mass balance for the solid phase and for the fluid in a fixed extraction bed. The model takes into account that a linear relationship describes the experimental data at equilibrium, the axial dispersion is negligible, and the solvent density and the flux along the bed are constant. The equations are solved numerically, and the parameters *k*_*TM*_, the global mass transfer coefficient (s^−1^), and *k*_*p*_, the volumetric partition coefficient of the extract between the solid and fluid phase at equilibrium, are determined by the parameter adjustment technique. This model has been successfully used in the representation of supercritical extraction data for different plant species and for different raw material structures considering different geometries [39,41,54,55]. The estimation of the parameters in all chosen models was performed by minimizing the sum of the squared errors between the average values obtained experimentally in triplicate and via mathematical models. The software used to estimate the parameters was Matlab^®^ and EMSO [56].

### 3.6. Analysis of the Extracts

The extracts obtained were solubilized in dichloromethane and applied to a silica gel 60 plate for analysis by TLC. The mobile phase used consisted of chloroform and methanol in a 98:2 ratio. After elution, the plate was visualized under UV light at a wavelength of 365 nm.

The samples were also analyzed by ultrafast liquid chromatography (UFLC) carried out according to the methodology developed by Medeiros-Neves [1]. The dry extracts were treated with acetone followed by filtration with filter paper to remove the waxes. Acetone was removed by evaporation under reduced pressure. Subsequently, an aliquot of approximately 2.5 mg of extract was dissolved in 1 mL of acetonitrile with the aid of ultrasound and diluted in an aqueous solution of acetonitrile (1:1) to 10 mL. The sample was filtered again through a filter suitable for injection into the chromatograph (2.2 μm). The equipment used was a Shimadzu SPD-M20A equipped with a diode array detector (DAD). Monitoring and processing of the output signal were performed by Shimadzu LC-solution Multi-PDA Software (LC-solution Version 1.25 SP4). A Shim-pack XR ODS column with a length of 100 mm, an internal diameter of 2.0 mm, and a particle size of 2.2 μm and a C18 SecurityGuard™ ULTRA pre-column (Phenomenex, Torrance, CA, USA) were used. The composition of the mobile phase used was 0.1% formic acid (v/v) and acetonitrile. The injection volume was 5 μL, the flow was set at 0.55 mL/min for 8 min, and the analysis temperature was set at 55 °C. The wavelength was set to 327 nm.

### 3.7. Cell Cytotoxicity by MTT Assay and Statistical Analysis

Cell viability assessment was carried out at the Laboratory of Applied Pharmacology (LAFAP) within the School of Health and Life Sciences at PUCRS utilizing the MTT colorimetric method. T24 cells were plated at a density of 5 × 10^3^ cells per well on a 96-well plate and left to incubate for 24 h. Subsequent to the incubation period, the cells underwent treatment with various concentrations of extract acquired through supercritical fluid extraction (ranging from 6 to 200 µg.mL^−1^) for 24 h. Post-treatment, the culture medium was removed, cells were rinsed with PBS (pH = 7.2–7.4), and MTT solution (100 μL) was added, followed by an incubation period of 3 h. Formazan crystals were dissolved in 100 μL of DMSO, and the optical density was gauged at 570 nm utilizing a SpectraMax Plus plate reader. The absorbance value was linearly correlated with the number of viable cells exhibiting active mitochondria. Outcomes were articulated as the percentage of absorbance in treated cells in comparison to controls. Relative cell viability was expressed as a percentage (%) relative to untreated control cells.

The statistical analysis employed was a one-way analysis of variance (ANOVA), followed by the Tukey post hoc test. Findings are depicted as the standard error of the mean. GraphPad Prism 8.0^®^ software was employed for graphical representation. *p* values below 0.05 were deemed indicative of statistical significance.

## 4. Conclusions

The optimal conditions found for supercritical extraction performance with regard to global yield were 240 bar and 60 °C for the operating conditions of the extraction vessel and microwave pretreatment temperature of 30 °C. The RSM analysis indicated pressure and temperature to be the variables of greatest significance on yield. The effect of the temperature applied in the microwave treatment was not statistically significant. The mathematical models used to simulate mass transfer were adequate and showed high adherence to experimental data. Model 2 performed slightly better than the other models. Both models 2 and 3 assume that external resistance is negligible, which suggests that transport of the extract within the plant material is difficult to attain. Although microwave pretreatment was not significant, steps from other types of pretreatment methods would be important to investigate to improve internal mass transfer. The analyses of the extracts allowed the characterization of the compounds 7-(2,3-epoxy-3-methyl-butyloxy)-6-methoxycoumarin, 5-(2,3-epoxy-3-methylbutyloxy)-6,7-methylenedioxycoumarin, 7-(3-methyl-2-butenyloxy)-6-methoxy-coumarin, and 5-methoxy-6,7-methylenedioxy-coumarin, the latter with known cytotoxic activity against glioma tumors. The extract rich in coumarins, obtained through supercritical fluid extraction, demonstrated cytotoxicity for a bladder tumor cell lineage (T24). These results corroborate previous research on the cytotoxic potential of coumarins identified against cancer cells. The originality of this study lies in the application of the circular bioeconomy concept in the processing of aerial parts of *P. polystachyum*, resulting from the use of steam distillation process residue as raw material to obtain coumarins. This research also concludes that it is viable to obtain two products, essential oil by steam extraction and coumarin-rich extract by supercritical extraction, using the same raw material, as well as enabling the introduction of a green technology, CO_2_ supercritical extraction, replacing the use of organic solvents, to obtain non-volatile extracts with cytotoxic activity against cancer cells.

## Figures and Tables

**Table 1:** ANOVA of the model generated by regression by the stepwise method.

	DF	Contribution	Adj SS	F	*p*
Model	6	85.08%	12.354	7.6	0.006
Linear	3	61.58%	17.883	11	0.003
PE	1	46.59%	40.590	24.98	0.001
TE	1	12.79%	11.139	6.85	0.031
TP	1	2.20%	1.921	1.18	0.309
Square	1	9.20%	8.016	4.93	0.057
TE^2^	1	9.20%	8.016	4.93	0.057
Interaction	2	14.30%	6.229	3.83	0.068
PE × TE	1	6.18%	5.382	3.31	0.106
PE × TP	1	8.12%	7.076	4.35	0.07
Residual error	8	14.92%	1.625		
Lack of fit	6	13.33%	1.935	2.79	0.288
Pure error	2	1.59%	0.695		
Total	14	100.00%			

DF: degrees of freedom; Adj SS: adjusted sum of squares; F: F-statistics; *p*: *p*-value. TE, PE, and TP correspond to extraction temperature, extraction pressure, and microwave pretreatment temperature, respectively.

**Table 2:** Parameters of the mathematical models for supercritical fluid extraction in optimal conditions.

Model 1			
D × 10^11^ (m/s^2^)	kc × 10^7^ (m/s)		R^2^
4.07	2.04		0.9830
Model 2			
$M^{*}$ (g)	τ (s)	ks × 10^8^ (m/s)	R^2^
1.1	307	3.55	0.9932
Model 3			
K_TM_ × 10^3^ (s^−1^)	k_p_ × 10^3^ (m^3^/kg)		R^2^
4.016	6.989		0.9524

**Table 3:** Three-factor Box–Behnken planning.

Experiment	Extraction Temperature	Extraction Pressure	Pretreatment Temperature
1	50	160	30
2	60	160	90
3	50	160	150
4	40	160	90
5	60	200	150
6	40	200	150
7	50	200	90
8	40	200	30
9	60	200	30
10	40	240	90
11	50	240	150
12	50	240	30
13	60	240	90
14	50	200	90
15	50	200	90

## Data Availability

Data are contained within the article.

## Supplementary Materials

The following supporting information can be downloaded at: https://www.mdpi.com/article/10.3390/molecules29122741/s1. Table S1: Results of experiments for Box-Behnken design.
