# Supplementary material for: Supercritical Carbon Dioxide Extraction of Coumarins from the Aerial Parts of Pterocaulon polystachyum
Source: Molecules. 2024 Jun 8;29(12):2741. doi: 10.3390/molecules29122741 (PMC11205997; doi:10.3390/molecules29122741)
Supplement: Supplementary file 1 [file molecules-29-02741-s001.zip › molecules-3015322-supplementary.pdf]

Table S1: Results of experiments for Box-Behnken design

Run	TE(°C)	PE(bar)	TP(°C)	Mass(g)	Yield(%)
1	50	160	30	0,94	4,07
2	60	160	90	0,72	3,11
3	50	160	150	1,71	7,44
4	40	160	90	2,56	11,12
5	60	200	150	1,02	4,44
6	40	200	150	1,60	6,96
7	50	200	90	0,93	4,06
8	40	200	30	1,90	8,26
9	60	200	30	0,77	3,33
10	40	240	90	2,14	9,30
11	50	240	150	0,82	3,57
12	50	240	30	0,97	4,23
13	60	240	90	1,18	5,14
14	50	200	90	0,82	3,57
15	50	200	90	0,89	3,87
